# Unravelling functional neurology: a critical review of clinical research articles on the effect or benefit of the functional neurology approach

**DOI:** 10.1186/s12998-018-0198-7

**Published:** 2018-07-25

**Authors:** Anne-Laure Meyer, Charlotte Leboeuf-Yde

**Affiliations:** 1 Complexité, Innovation et Activités Motrices et Sportives, Université Paris-Saclay, 91405 Orsay Cedex, France; 2 Complexité, Innovation et Activités Motrices et Sportives, Université d’Orléans, 45067 Orléans, France; 3 Institut Franco Européen de Chiropraxie, 24 Bld Paul Vaillant Couturier, Ivry sur Seine, 94200 Paris, France

**Keywords:** Functional neurology, Chiropractic, Critical review, Evidence, Effect, Benefit, Neurologie Fonctionnelle, Chiropraxie, Revue critique, Evidence scientifique, Effet thérapeutique, Bénéfice clinique

## Abstract

**Background:**

Functional Neurology (FN), mainly practiced by chiropractors, proposes to have an effect or a benefit on varied clinical cases, from debilitating diseases to performance enhancement in asymptomatic people.

**Objectives and design:**

A critical review of publications captured in and from the journal *Functional Neurology, Rehabilitation, and Ergonomics* (FNRE) was performed in order to investigate whether there is evidence on clinical effects or benefits of FN. This review had five research objectives, three relating to the type of literature available through this journal, and two in relation to design and methodological aspects of the included studies.

**Method:**

All issues of the FNRE journal were searched (October 2017), including a handsearch of their lists of other relevant publications. In order to find evidence in relation to the effect or benefit of FN, the search was restricted to prospective clinical research studies with a control group, claiming or appearing to deal with the topic. The review was undertaken by two independent reviewers using two checklists, one relating to study description, and one on quality. Results were reported narratively.

**Results:**

Nine articles were found. The FNRE journal contained 168 authored texts, of which 36 were *research studies* (21%). Four of these were *clinical research studies* on FN effect or benefit (2%). Another five were obtained through the handsearch. The included studies were conducted on adults or children, symptomatic or not, and investigated various interventions consisting of single or multiple stimuli, of varied nature, all primarily said to be provided to stimulate brain areas. Conditions included attention deficit disorders, attention deficit and hyperactivity disorders, autism-spectrum disorders, cortical visual impairment, traumatic brain injury, and migraine. Balance and the “blind spot” were investigated in healthy subjects. Major design and methodological issues were identified and discussed for all the nine studies; only four were considered as (potentially) appropriate for further scrutiny. However, these were of low methodological quality and, therefore, no robust evidence could be found in relation to the effect or benefit of the tested FN interventions.

**Conclusions:**

This journal contains no acceptable evidence on the effect or benefit of FN in relation to various conditions and purported indications for intervention.

## Resume

**Introduction:**

La Neurologie Fonctionnelle (NF), approche thérapeutique principalement pratiquée par des chiropracteurs, permettrait d’obtenir des effets ou bénéfices cliniques dans de nombreux cas, allant de pathologies handicapantes à l’amélioration des performances chez des personnes sans problème de santé.

**Design et objectifs:**

Une revue critique de la littérature des publications obtenues à partir du journal *Functional Neurology, Rehabilitation, and Ergonomics* (FNRE) a été conduite afin d’étudier s’il existe une évidence scientifique concernant les effets ou bénéfices cliniques de la NF. Cette revue avait cinq objectifs, trois en rapport avec le type de littérature disponible à travers ce journal, et deux en rapport avec les *designs* et aspects méthodologiques des études incluses.

**Méthode:**

L’ensemble des volumes du journal FNRE ont été consultés (octobre 2017), y compris les listes de références des publications faites par les membres de l’association *International Association of Functional Neurology and Rehabilitation*. Dans le but d’étudier les faits scientifiques concernant les effets ou bénéfices cliniques de la NF, notre recherche a été limitée à des études cliniques prospectives incluant au moins un groupe contrôle, annonçant ou paraissant traiter du sujet. La revue a été menée par les deux auteurs, de manière indépendante, afin d’extraire les informations descriptives et d’évaluer la qualité méthodologique des articles inclus. Les résultats ont été rapportés de manière narrative.

**Résultats:**

Neuf articles ont pu être inclus. Le journal FNRE contenait 168 textes avec mention d’au moins un auteur, parmi lesquels 36 étaient des études de recherche (21%). Seules quatre étaient des études cliniques portant sur l’effet ou le bénéfice de la NF (2%). Cinq autres études cliniques ont été obtenues via les listes de références mentionnées ci-dessus. Les neuf études incluses avaient été conduites sur des adultes ou des enfants, symptomatiques ou non, et investiguaient diverses interventions consistant en des stimuli, uniques ou multiples, de nature variée, tous présentés comme utilisés pour spécifiquement stimuler des régions cérébrales. Les conditions étudiées incluaient des troubles du déficit de l’attention avec ou sans hyperactivité, des troubles du spectre autistique, des cas de déficience visuelle d’origine corticale, de traumatismes crâniens, et de migraines. Des changements d’équilibre et de taille du « blind spot » ont été investigués chez des sujets sains. Des problèmes de *design* et de méthodologie ont été identifiés et discutés pour ces neuf études; seules quatre ont été considérées comme (potentiellement) appropriées pour une évaluation de leur qualité méthodologique. Ces dernières étaient de faible qualité méthodologique et, de ce fait, aucune évidence scientifique solide n’a pu être trouvée en relation avec l’effet ou le bénéfice des modalités thérapeutiques de NF testées dans ces études.

**Conclusion:**

Le journal FNRE ne contient pas d’évidence scientifique pouvant soutenir l’effet ou le bénéfice d’une approche thérapeutique en NF et ce, concernant diverses conditions et indications supposées.

## 抽象

**背景:**

主要由脊椎治疗师实施的功能性神经病学(FN)提议对各种临床病例(从衰弱疾病到无症状人群表现增强)产生效果或益处。.

**目标和设计:**

对功能性神经病学,康复和人体工程学(FNRE)期刊中收录的出版物进行评论性审查,以调查是否有FN临床效果或益处的证据。该评价有五个研究目标,其中三个与通过该期刊提供的文献类型有关,另外两个涉及所纳入研究的设计和方法学方面。.

**方法:**

搜索所有FNRE期刊(2017年10月),包括手工搜索其他相关出版物的清单。为了找到与FN的效果或益处相关的证据,该搜索仅限于与对照组进行的前瞻性临床研究研究,声称或似乎处理该主题。评审由两名独立评审员使用两份清单进行,一份与研究描述相关,另一份与质量相关。叙述性地报道了结果。.

**结果:**

发现了9篇文章。 FNRE杂志包含168篇着作文章,其中36篇是研究报告(21%)。其中四项是关于FN效应或益处的临床研究(2%)。另外五个是通过手工搜索获得的。所纳入的研究是针对成年人或儿童进行的,无论是否有症状,并研究了由单一或多重刺激组成的各种干预措施,这些干预措施具有不同的性质,所有这些主要都是为了刺激大脑区域。症状包括注意力缺陷障碍,注意力缺陷和多动症,自闭症谱系障碍,皮质视觉障碍,创伤性脑损伤和偏头痛。平衡和“盲点”在健康受试者中进行了调查。主要的设计和方法论问题在所有九项研究中都得到确认和讨论;只有四个被认为(可能)适合进一步审查。然而,这些方法的质量很低,因此,没有有力的证据可以发现与测试的FN干预措施的效果或益处有关。.

**结论:**

本期刊没有关于FN对各种症状和干预适应症的影响或益处的可接受证据。.

## Background

Functional Neurology (FN), a therapeutic approach founded by a chiropractor, FR Carrick, proposes treatment to alleviate many chronic and even incurable conditions [1]. Given the diversity of symptoms and disorders that “functional neurologists” claim to deal with, ranging from musculoskeletal to neurodegenerative, this approach would have the potential to improve the quality of life of many people.

Therefore, FN interests many practitioners [2, 3], including chiropractors, a profession in which this approach may well be spreading. This is mainly achieved through seminars given by private organizations such as the *Carrick Institute*. The offer of FN seminars is sizable and it is necessary to attend many of them before reaching a certain level of proficiency as a “functional neurologist” [4].

FN is based on the assumption that reversible lesions in the nervous system, especially in the brain, are the cause of a multitude of conditions and that specific clusters of deficient neurons (e.g. neurons on one side of the cerebellum) can be positively affected by various stimuli, including but not restricted to manipulative therapy [1, 5].

For example, in a book chapter dedicated to clinical cases, the author describes the FN approach of an attention deficit and hyperactivity disorder (ADHD) patient as well as of a case of low back pain with spinal root compression [6]. In the first case, a FN diagnosis of right cerebral hemisphere and left cerebellum dysfunctions was made. The second case led to a FN diagnosis of right hemisphere dysfunction, meaning that for two such very different types of conditions an almost identical FN diagnosis may be provided. Both cases were treated in the manner of FN with joint manipulations, breathing exercises and nutritional support. The only difference was that, for the case of ADHD, treatment was complemented with sound therapy and spatial rearrangement exercises.

This approach does not appear to be generally accepted in classical neurology, and although many, if not most, of the diagnostic tools used in FN are also known in classical medicine, the interpretation made by “functional neurologists” is probably not always known or understood outside FN, such as their use of the “blind spot” [7], which has evoked questions and criticism [8–13].

Further, a recent scoping review on the topic of FN [1] found that despite the extensive list of supposed FN indications, only a few of these appear to have been described in the scientific literature, with an apparent lack of studies in relation to treatment effect, i.e. a lack of studies with robust design. However, this scoping review investigated FN in a context of chiropractic manual therapy and it is therefore possible that evidence might exist on FN interventions that do not include an element of manual therapy (which is not systematically used by “functional neurologists”), for which reason this area merits further investigation.

Many chiropractors are using, at least, elements of FN on their patients, but as the chiropractic profession in many countries is legally recognized and hence expected to be evidence-seeking and evidence-accepting, it is important to investigate the level of such evidence regarding FN, including its clinical effect or benefit. Such evidence is required for FN to be regarded as credible. Obviously, this requirement would be the same for any therapeutic approach when, as in this case, it is not an accepted part of mainstream medicine, and in particular if the theories on which they rest are not generally considered to be plausible in the light of present-day state-of-the-art knowledge.

Unfortunately, scientific literature that covers FN topics has already been found by the present authors to be difficult to capture, making a systematic search difficult when searching for indexed literature in scientific databases. One of the most well-known authors within FN previously directed us to the journal *Functional Neurology, Rehabilitation, and Ergonomics* (FNRE) (G Leisman, personal communication). This journal is published by the Nova Science Publishers group [14], not indexed in Medline or Scopus and therefore somewhat difficult to find. It is affiliated to the *International Association of Functional Neurology and Rehabilitation*, an FN organization promoting this approach, including through research activities such as the FNRE journal.

Although this journal states under its “aim and scope” to deal with topics other than FN, a list of neurological disorders is provided with diseases and traumatisms of the brain listed first under its “description of the field covered”. Therefore, this appears to be a major source of information on the FN approach. The FNRE journal was previously searched for its scientific contents in relation to FN in a chiropractic context but resulted in only three relevant articles [1]. Nevertheless, relevant information on FN in general might still be found in this journal, as manipulation is only one of the therapeutic tools available to “functional neurologists”.

We therefore decided to perform a critical review of all publications in this journal to investigate the evidence for clinical effect or benefit of FN. Specifically, our research objectives were:

1-To define the proportion of articles that are *research studies* (i.e. not narrative reviews, discussion papers, poster abstracts, abstracts, editorial material, or public relation information) in the FNRE journal.

2-To define how many of those are *clinical research studies* that purported or appeared to deal with effect or benefit of FN.

3-To describe which indications and FN interventions were studied in the *clinical research studies* captured through the FNRE journal.

4-In these studies, to establish whether the design and overall study method were suitable for research into the effect or benefit of FN.

5-To describe the evidence available in relation to the clinical effect or benefit of FN, taking into account some minimal methodological criteria.

## Methods

### Search strategy for information and screening procedure

All issues of the journal *Functional Neurology, Rehabilitation, and Ergonomics* were obtained in October 2017. At this period, all of its six volumes and twenty-four issues, edited between 2011 and 2016, were searched for *research studies* on FN effect or benefit, based on titles and abstracts, and, when needed, on full texts. No issues were published before 2011 or after 2016, at the time of writing this report (December 2017 to January 2018).

The texts in these issues were blindly screened by the authors, according to predetermined definitions of which articles would be considered acceptable, i.e. considered as *research studies* (defined in the section *Inclusion and exclusion process of articles*). Thereafter, the two authors extracted from all the research articles those articles that were *clinical research articles* reporting on effect or benefit of FN.

Most issues of the journal FNRE contain a section entitled “*IAFNR News and Events”,* where various types of information about the *International Association of Functional Neurology and Rehabilitation* and its members is reported. Within this information, lists of recent publications in peer-reviewed journals authored by members of the *International Association of Functional Neurology and Rehabilitation* were available. These reference lists were independently searched by the authors to find additional *clinical research studies* in relation to effect or benefit of FN. Only titles of published or scientific articles accepted for publication were considered in these lists, which mixed published, accepted, and submitted articles, as well as conference papers.

### Inclusion and exclusion process of articles

*Step 1*: In order to define the proportion of articles that were *research studies* in the six volumes and twenty-four issues of the journal FNRE, the total of *texts* was counted twice by ALM on the basis of the table of contents of each issue. We defined as a *text* a written script introduced by a title for which at least one author’s name was mentioned. Therefore, *texts* may include written scripts as varied as scientific articles, abstracts, editorials, and letters to editor.

Articles were considered as *research studies* when they had 1) one or several research questions or research objectives and 2) a methods section that explained the process of data collection and data analysis. This means that discussion papers, narrative reviews but also case reports would not be counted as such. We did not include research information presented solely in abstracted form, such as posters and conference proceedings, as they do not contain full information of the study project. At this stage, we did not differentiate experimental studies from clinical studies and we also included studies that dealt with other topics than FN.

*Step 2*: *Research studies* were included as *clinical research studies* dealing with effect or benefit of FN if 1) the intention to investigate an effect or benefit was obvious, searching throughout the articles for words such as “effect(s)”, “effectiveness”, “improvement(s)”, “improve”, “recover”, “recovery”, or “benefit(s)”, and 2) the intervention that was investigated had the hallmark of FN, as it was described in a previous review [1]. Furthermore, *research studies* were considered as *clinical* when the investigated intervention was clearly known or identified as already used in clinical practice, specifically within FN.

Any intervention that included a stimulus said to be directed to the nervous system could be included, given that the FN approach had been previously described as consisting of almost any kind of stimulus purported to stimulate the nervous system, especially the brain [1]. This could also be multifaceted, i.e. consisting of various stimuli, being or not complemented by nutritional counseling or supplements.

These studies could report results on symptomatic or asymptomatic subjects and, if subjects were symptomatic, symptoms could be of any kind, according to the wide supposed scope of FN.

Hence, at this step, any full text *clinical research studies* regarding FN effect or benefit could be included, regardless their study design, i.e. appropriate or not to investigate an intervention effect or benefit but case reports, narrative reviews and discussion papers were not included for the reasons explained above. Also, articles were included regardless of the type of subjects and FN intervention investigated.

*Step 3*: For further scrutiny, we searched for prospective studies with at least one control group.

We selected those studies that related to the *effect* of an intervention, if it was investigated in one of two ways: the intervention could be compared 1) to a sham procedure (to control for the placebo effect), or 2) to an intervention already known to be effective, i.e. already tested against placebo.

Also prospective studies with other types of control groups (e.g. control group subjected to an intervention accepted in medical practice for the investigated condition) were included in order to investigate *benefit* of intervention. Studies could be included whether they were conducted or not with a random allocation.

Retrospective studies and studies without one or several control groups were not considered suitable at this step.

### Extraction of information

Two checklists were created for the review: one related to the description of the studies (Table 1) and one to their methodological quality (Table 2). The latter consisted of two parts. The first part concerned all the *clinical research studies* included and contained only one item in relation to the design of the study and its potential appropriateness to investigate an effect or benefit.

**Table 1:** Descriptive checklist of eight *clinical research studies* plus one clinically relevant *research study* on Functional Neurology approach included in a critical review

1st Author Year Journal	Topic covered	Study subjects:-Type -Age (range) -Origin -Number (males/females)	-Intervention -Control (other than sham) -Sham	-Outcome -How was it assessed?	When was it assessed?	Ethics approval? (with a clear mention of its origin)	Conflict of interest (reported or supposed)
Malkowicz 2006 [20] Intern J Neuroscience	Cortical visual impairment	Intervention group -Pediatric patients diagnosed with cortical visual impairment −13-120 months -Selected from a clinical database −21 (?/?) Control group -Patients diagnosed with cortical visual impairment -? -Unclear − 67 (?/?)	-Individualized at-home *intensive visual program*, during 4–15 months -Retrospective control sample from a previous published study, probably on the natural course -None	-*Visual level* -*Developmental profile* (an evaluation tool, proper to the clinic from where the patients were recruited, which included a visual scale)	-Before -Follow-ups at least every 6 months if treatment lasted that long (only for intervention group, no exact time of follow-up(s) was given for the external control group)	Unclear	No mention about any potential conflict of interest
Daubeny 2010 [21] Int J Disabil Hum Dev	Brain function	Intervention group -Healthy adults -(?-?) -? − 31 (?/?) Control group -Healthy adults -(?-?) -? − 31 (?/?)	−10 upper extremity manipulations -None -Upper extremity sham manipulations with unloaded activator instrument	-*Blind-spot* size -*Blind-spot* measurement	-Before -Immediately after	Unclear	Authors reported to have no competing interests However, at least 2 authors are known to have business interest in relation to the topic.
Leisman 2010a [22] Int J Disabil Hum Dev	Attention-deficit hyperactivity disorders	Intervention group 1 -Children with ADHD − 6-11 years -Several clinics − 36 (36/0) Intervention group 2 -“Normal” children -Age-matched with the ADHD group -? − 15 (15/0) Control group 1 -Children with ADHD − 6-11 years -Several clinics − 42 (42/0) Control group 2 -“Normal” children -Age-matched with the ADHD group -? − 16 (16/0)	-*Motor sequencing training*, 3-month course -No *motor sequencing training* -None	-*Signal detection* performance -*Signal detection* task	-Before -After	No information was found	No mention about any potential conflict of interest However, at least 1 author is known to have a business interest in relation to the topic.
Leisman 2010b [23] Int J Adolesc Med Health	Attention deficit-disorders/Attention-deficit hyperactivity disorders	-Child patients with ADD/ADHD −6-12 years -Clinics (said to be associated with one of the authors) −122 (94/28)	−12-weeks individualized *hemispheric specific remediation program*, 3 times/week, 1 h each (i.e. 36 sessions) -None -None	1- *Sensory and motor function* 2- Academic performance 3- Behaviors 1- *Functional assessments of sensory and motor function* 2- Wechsler Individual Achievement Tests 3- Brown Attention Deficit Disorders Scales	-Before -After	No information was found	Yes, 1 is reported: Patients came from clinics where 3 of the authors have financial interest in the topic. In addition, the project was funded by an institution with known financial interest in the area.
Carrick 2011 [16] Funct Neurol Rehabil Ergon	Balance	-Adults −24-52 years -? −25 (16/9)	-Whole body rotation over 40 s -None -None	-*Stability* and *sway* -Dynamic computerized posturography system (CAPS™ Professional System)	-Before -After	Unclear	Yes, 1 is reported: Two authors “are currently employed and are part owners of the Vestibular Technologies, LLC” In addition, the project was funded by an institution with known financial interest in this area.
Castellanos 2012 [24] Funct Neurol Rehabil Ergon	Stated in title: traumatic brain injury but according to Methods: stroke	Intervention group -Adult patients with traumatic brain injury − 18-51 years -? − 15 (?/?) Control group -Healthy volunteers age and gender-matched -Age-matched with the intervention group -? − 14 (?/?)	-Individualized neuropsychological rehabilitation, 3–4 times/week for 1 h/session, during 7–12 months +/− associated with physiotherapy, speech therapy, and/or occupational therapy -“Control group” at baseline only (healthy subjects were not subjected to any intervention) -None	-Complexity and entropy of brain activity -Magneto-encephalography	-Before -After (only for intervention group, control group was assessed only at baseline)	Unclear	No mention about any potential conflict of interest
Carrick 2013 [17] Funct Neurol Rehabil Ergon	Balance	Study 1: -Healthy adult volunteers −20-60 years -Recruited from advertisements −52 (31/21) Study sample was randomly allocated to 4 groups, details regarding age (range) and gender were not given for each of them. Study 2: -Healthy adult volunteers − 20-61 years -Recruited from advertisements − 56 (33/23) Study sample was randomly allocated to 4 groups, details regarding age (range) and gender were not given for each of them.	Both studies: -Whole body rotation over 40 s for all groups -Each group (4 per study) differed in terms of pitch and yaw planes during whole body rotation -None	Study 1: -Eight posturographic measures Study 2: -Six poturographic measures Both studies: -Dynamic computerized posturography system (CAPS™Professional System)	Both studies: -Before -Immediately after − 1 day after −1 week after	Unclear	No mention about any potential conflict of interest However, at least 1 author is known to have a business interest in relation to the topic. Another 2 have/had a financial interest in the posturography equipment [16].
Sullivan 2013 [18] Funct Neurol Rehabil Ergon	Migraine	Intervention group -Female adult patients or volunteers, all in midst of a migraine attack − 15-53 years -Referred from local medical clinics or recruited from advertisements − 13 (0/13) Control group -Female adult patients or volunteers, all in midst of a migraine attack − 25-38 years -Referred from local medical clinics or recruited from advertisements − 3 (0/3)	-Pneumatic ear insufflation, provided in roughly 30s intervals with a minimum of 3 insufflations -None -Otoscope with insufflation speculum with no pneumatic pressure applied	-Pain -Visual analog scale	-Before -During (after each insufflation) -30 min after -4 h after -24 h after	Unclear	No mention about any potential conflict of interest
Bousquet 2015 [19] Funct Neurol Rehabil Ergon	Attention-deficit hyperactivity disorders /Autism-spectrum disorders	-Student volunteers with ADHD or ADS (identified with a “right-hemisphere weaknesses”) -*Hemisphere integration therapy* tutoring center − 7-16 years − 12 (10/2)	-Individualized *hemisphere integration therapy*, 36 individual/group sessions, 3 times/week, 1 h each, combined with nutritional training and home exercises -None -None	1-Self-perception of academic, sensory, and motor abilities 2-Behavior 3-Cognitive skills 4-Senrory and motor skills 1-Semi-structured interviews 2-Brown Attention Deficit Scales / Gilliam Autism Rating Scales / Gilliam Asperger’s Syndrome Scales 3-Wechsler Individual Achievement Test III 4-Perdue Pegboard performance / Dichotic Word Listening Test / Aerobic, core and balance exercises	-Before -After	No information was found	No mention about any potential conflict of interest

*ADHD* Attention deficit and hyperactivity disorders, *ADD* Attention deficit disorders, *ASD* Autism-spectrum disorders

**Table 2:** Quality checklist of eight *clinical research studies* plus one clinically relevant *research study* on Functional Neurology approach included in a critical review

1st Author Year Journal	All studies included		*Clinical research studies* with appropriate or potentially appropriate study design to investigate an effect or benefit of Functional Neurology approach						
	-Design -Design appropriate to investigate effect or benefit of intervention?	If design was not appropriate, major methodological considerations (“NA” for appropriate or potentially appropriate study design)	Were study subjects stated to be: -Blind to treatment allocation? (NA if no sham) -Naïve to types of intervention?	-Was a random allocation reported? -Was it stated that this was concealed? (NA if no random allocation)	Were interventions well described?	Was the assessor reported to be blind?	Outcome measure reported to be reliable or reproducible?	Was the person who analyzed the data stated to be blind?	Were losses or exclusions reported or obvious in results, tables or graphs?
Malkowicz 2006 [20] Intern J Neuroscience	Retrospective study of clinical database with external control group (from previously published study) -No	In order to investigate the effect of intervention, it would be necessary to include a concomitant control group to ensure that the two groups are similar and assessed at similar interval(s).							
Daubeny 2010 [21] Int J Disabil Hum Dev	-Randomized controlled trial -Yes	NA	-No -No	-Yes -No	Yes	Yes	No	No	Yes
Leisman 2010a [22] Int J Disabil Hum Dev	-Two randomized controlled trials (?) -Yes	NA	-NA -No	-Yes -No	Yes	No	No	No	No
Leisman 2010b [23] Int J Adolesc Med Health	-Case series from multiple clinics (?) or Multicenter outcome study (?)* -No	In order to investigate an effect, a control group would be needed.							
Carrick 2011 [16] Funct Neurol Rehabil Ergon	-Outcome study -No	In order to investigate an effect or benefit, a control group would be needed.							
Castellanos 2012 [24] Funct Neurol Rehabil Ergon	-Outcome study with healthy untreated control group at baseline -No	In order to investigate effect or benefit, a similar control group subjected to another intervention would be needed.							
Carrick 2013 [17] Funct Neurol Rehabil Ergon	-Four arms randomized trial (?) -Potentially	NA	-NA -No	-Yes -No	Yes	No	No	No	No
Sullivan 2013 [18] Funct Neurol Rehabil Ergon	-Prospective case series with sham treatment in 3/13 cases -Potentially	NA	-No -No	-No -NA	Yes	No	No	No	Yes
Bousquet 2015 [19] Funct Neurol Rehabil Ergon	-Outcome study -No	In order to investigate an effect, a control group would be needed.							

NA: Not applicable

(?): Uncertainty

*Reported as a pilot study by the authors but used for making conclusion about the effect of the FN intervention. Also the fact that this was a multicenter study was not clear

If the design was not considered appropriate, from a methodological perspective, remedial propositions were given in order to promote the conduct of studies that would be able, in term of study design and methodology, to investigate whether the FN approach has an effect or benefit.

If the study design was considered potentially appropriate to study effect or benefit of an intervention, the article was reviewed for further quality assessment, based mainly on some items proposed in the Cochrane recommendations [15]. For this we developed a seven-component quality checklist consisting of five risk-of-bias items, one item relating to external validity and one to unsystematic methodological errors as described in Appendix. Sometimes, other glaring methodological problems were mentioned in the text.

These items were added up for each article and percentages calculated taking into account the possibility of the occasional item being irrelevant (not applicable). No cut point was set for acceptability but the final score was instead used to illustrate, in a very basic way, the level of scientific rigor and credibility of the included articles.

All selected articles were reviewed independently and blindly by the two authors. Information was sought throughout the text but not in the abstract and discussion sections. Data collected in the two checklists by the two authors were compared and discrepancies resolved by consensus.

Initially, a third checklist, related to the results of the effect or benefit studies with the most robust designs was considered but finally not needed, as will be evident further in this report.

### Data synthesis

**Fig. 1:**
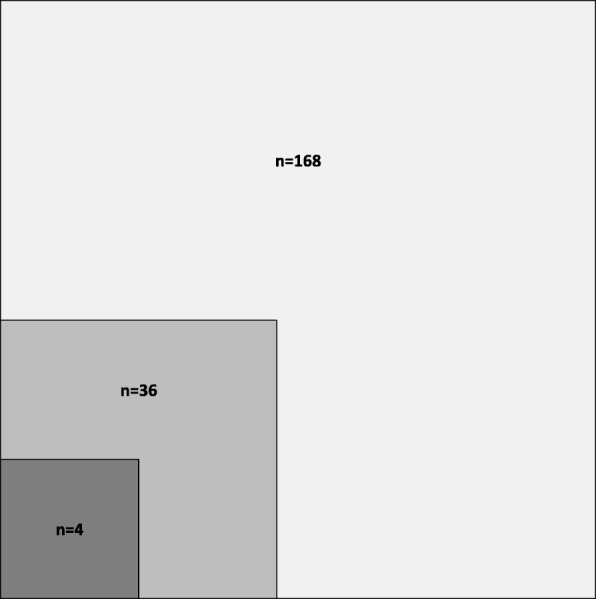
Proportion of *research studies* and *clinical research studies* of all texts published in six volumes of the journal *Functional Neurology*, Rehabilitation, and Ergonomics

The results of the selection process (Fig. 1) served to provide information for our two first research objectives. Tables 1 and 2 were created for the remaining research objectives. In both tables, articles were presented consecutively by year of publication. On their basis, a narrative synthesis of the collected data was provided for each research objective.

## Results

### *Proportion of research studies and clinical research studies dealing with effect or benefit of Functional Neurology* (research objectives 1 and 2)

As illustrated in Fig. 1, in a total of 168 texts found in the journal *Functional Neurology, Rehabilitation, and Ergonomics*, 36 were identified as *research studies* (clinical and experimental). Among them, four were identified as *clinical research studies* dealing with effect or benefit of FN [16–19]. This means that 21% of the texts contained in its six volumes, published from 2011 to 2016 are *research studies* in general, and 2% of all texts are *clinical research studies* on the effect or benefit of the FN approach.

One of the *research articles* (Castellanos et al. [24]) was defined as not dealing with FN effect or benefit but was nevertheless reported on because it dealt with other relevant clinical issues. This scientific article was counted as a *research study* but not as a *clinical research study*, which would not change substantially the percentage of the latter. Nevertheless, for the sake of interest, it will be described with the other studies, resulting in five relevant articles.

The handsearch in the section “*IAFNR News and Events”* of the FNRE issues provided four additional *clinical research studies* in our area of interest [20–23], all from other scientific journals. A total of eight *clinical research studies* on FN effect or benefit were therefore included in the review in order to fulfill our three other research objectives, plus the additional clinically relevant study [24], bringing the number to nine.

Study objectives were not always clearly stated, and if (as was sometimes the case) the introduction was also unstructured and confusing, the whole text had to be scrutinized to identify the purposes of the studies. For this, we searched for terms such as “effect” and “effectiveness” in the texts. As shown in Table 3, the intention to investigate an effect or some type of benefit of FN was clearly stated by the authors in eight of the included studies. The ninth (Castellanos et al. [24]) appeared to us first as having the same intention (as shown in Table 3), however, after further scrutiny and discussions, it was not considered to intend to investigate treatment effect or benefit of FN.

**Table 3:** Table illustrating the respective authors’ intention to study the effect or benefit of Functional Neurology approach

1st Author Year Journal	Signs that authors intended to study effect or benefit (non-exhaustive list of concerned article sections and examples, limited to two examples per publication)
Malkowicz 2006 [20] Intern J Neuroscience	-Introduction/Objective “…the authors were particularly interested in studying the effects of an intensive visual stimulation treatment program on visual recovery.” (p.1018) -Discussion “…it can be seen that visual stimulation programs improve a brain-injured child’s ability to see significantly more than that of an individual not receiving visual stimulation.” (p.1032)
Daubeny 2010 [21] Int J Disabil Hum Dev	-Title “Effects of contralateral extremity manipulation on brain function” -Discussion “The Sham manipulation did not have such an effect supporting that observations that it is the manipulation itself that is causing the changes in brain function.” (p. not available)
Leisman 2010a [22] Int J Disabil Hum Dev	-Title “Effects of motor sequence training on attentional performance in ADHD children” -Abstract “Rhythm feedback training appears to have a significant effect on clinically observed changes in behavior in attention-deficit/hyperactivity disorder…” (p.275)
Leisman 2010b [23] Int J Adolesc Med Health	-Title “The effect of hemisphere specific remediation strategies on the academic performance outcome of children with ADD/ADHD” -Discussion “We here attempted a pilot study to determine if treatment that is preferentially aimed at a hypothesized interactive right hemisphere in ADD/ADHD children would have an effect on their sensory motor performance, as well as on cognitive function related to attention focus.” (p.281)
Carrick 2011 [16] Funct Neurol Rehabil Ergon	-Title “The effects of whole body rotations in the pitch and yaw planes on postural stability” -Results “To investigate the effects of the Rotation, one tailed t-test for paired observations with…” (p.174)
Castellanos * 2012 [24] Funct Neurol Rehabil Ergon	-Title “Restoring the brain entropy and complexity after rehabilitation of traumatic brain injury” -Discussion “After rehabilitation, the local networks recover, understanding recovery as an approach to control values of organization.” (p.212)
Carrick 2013 [17] Funct Neurol Rehabil Ergon	-Title “The effect of off vertical axis and multiplanar vestibular rotational stimulation on balance stability and limits of stability” -Method “To evaluate the effects over time the rotational stimulation could have on the balance of the subjects, each…” (p.347)
Sullivan 2013 [18] Funct Neurol Rehabil Ergon	-Introduction/Objective “Our task was to investigate the effectiveness of this simple, non-invasive, low-cost and readily available bedside therapy.” (p.94) -Discussion “…, it would seem unlikely that the observed effects are due particularly to stimulation of…” (p.102)
Bousquet 2015 [19] Funct Neurol Rehabil Ergon	-Title “The perceived effects of hemisphere integration therapy on students with identified right hemisphere weakness” -Conclusion “Before this study, there was no research regarding the effects of HIT on students with ASD.” (p.292)

*This article was later considered not to deal with effect/benefit

### *Description of clinical research studies purported to investigate the effect or benefit of functional neurology*

A. **General description**

The nine included articles were published between 2006 and 2015, five in the journal *Functional Neurology, Rehabilitation, and Ergonomics*, two in the *International Journal on Disability and Human Development*, one in the *International Journal of Neuroscience* and another in the *International Journal of Adolescent Medicine and Health*. Some authors had contributed to several of these articles: G Leisman co-authored five, and both FR Carrick and R Melillo co-authored four each.

None of the authors reported explicitly having an ethics approval from an identified ethics committee with an identification number of the application and approval (Table 1, col.7). Nonetheless, one reported to have ethics approval, two reported to have an ethics approval from an unidentified review board, which may or may not be the same as an official human research ethics committee, and another reported to be “approved” without more information. The remainder (*n* = 5) either mentioned that they acted in accordance with some ethical recommendations or provided no information.

As for conflict of interest, this was not mentioned at all in six of the reports, whereas two declared to have such a conflict and one declared to have none. Nevertheless, in the latter and in two of the “undeclared” articles, we identified potential conflicts of interest and, in the two “declared”, we identified some additional potential conflicts of interest (Table 1, col.8).

The choice of study design to investigate *effect* or *benefit* was often not respected and, in those studies where a full methodological assessment was done, the quality scores were low, never reaching 50%.

B. **Description in relation to indications and Functional Neurology interventions studied (Research objective 3)**

### Indications studied

The selected studies included either symptomatic (*n* = 6) or asymptomatic (*n* = 3) subjects: adults (*n* = 5) and children (*n* = 4) (Table 1, col.3). Study samples ranged from 12 to 122 subjects, control subjects included (when a control group was present) (Table 1, col.3).

Three studies reported on subjects diagnosed with ADHD (Table 1, col.2). One of them included also subjects diagnosed with attention deficit disorders and another reported on subjects diagnosed with ADHD or with autism-spectrum disorders. These two studies mixed these types of subjects on the basis that they were supposedly identified as having one brain hemisphere deficient compared to the other, referring to the FN concept of hemisphericity [25]. Two studies had balance as its main topic, which was investigated on healthy subjects without balance or gait disorders (Table 1, col.2 & 3). The last four studies reported on: i) cortical visual impairment, ii) brain function asymmetry in healthy subjects, iii) traumatic brain injury in, apparently, post-stroke subjects, and iv) migraine in people having a migraine episode (Table 1, col.2 & 3).

### Interventions studied

While the intervention was well described by most of the authors, in two articles this was poorly reported, making it difficult to fully understand what the FN approach contained [22, 24]. The FN interventions consisted of a single modality (*n* = 5) or were multifaceted and individualized (*n* = 4) (Table 1, col.4). Manipulative therapy (*n* = 1), motor sequencing training (*n* = 1), whole body rotation (*n* = 2), and pneumatic ear insufflation (*n* = 1) were tested as single modalities of intervention. Multifaceted programs were of different kinds, consisting of a combination of visual stimuli (*n* = 1), of a neuropsychological rehabilitation program, complemented or not by one or several physical modalities (*n* = 1), or of a combination of mainly motor, sensory and cognitive stimuli (*n* = 2).

The total numbers of treatment sessions and their frequency were disparate, ranging from a single intervention of 40 s to 4–15 months of a home program, which probably involved daily stimuli (Table 1, col.4). Outcome measures used to assess effect or benefit also varied from one study to another but all assessed them at least before and after intervention, at various time points (Table 1, col.5 & 6).

### *Consideration of major design and methodological issues* (research objective 4)

All studies included in this review had major design and/or methodological problems in relation to study the effect or benefit of FN. In this section, the main issues that we identified are reported.

A. **Study designs unable to detect effect or benefit of interventions**

### Studies without control group

Two articles reported on outcome studies (Carrick et al. [16], Bousquet [19]) and one on an outcome study or on a case series (it was unclear if this was a prospective or a retrospective study) (Leisman et al. [23]). Unlike reported by the authors of this study, we did not consider it as a pilot study. The reason is that a pilot study may be used for several reasons before conducting a clinical trial but in such study it is not suitable to test clinical hypotheses and provide estimates of effect [26], which was the case in the article of Leisman et al. [23]. These three articles did not include a control group and were therefore not included in our final analysis.

### Study with control group that was not concomitant

Malkowicz et al. [20] had as their research objective to study “the effects of an intensive visual stimulation treatment program on visual recovery” in children diagnosed with cortical visual impairment. This study consisted of a retrospective analysis of a clinical database with an external control group from a previously published study and was not included in our final analysis. The reasons for this were that this study lacks two important aspects: (i) study subjects were not included in the study at about the same time, meaning that the disorder and treatment may have become different over time, and (ii) as the intervention-and control- groups were not included under the same circumstances, it is likely that they were not comparable on all or most variables, apart from the tested intervention. This makes it difficult to ensure that it is the treatment that matters and not some other circumstances.

Nevertheless, the design of this study would be suitable to provide preliminary insights into a rare condition with poor prognosis. However, in the present study, since the authors state that “time is the factor of essence”, it would have been important, when comparing results in two different groups, that the visually impaired children were all at the same stage of the disease and assessed at similar time intervals, which is not clear in this report. The results may well be encouraging, for which reason this study could be considered as a first step to inspire a proper randomized controlled trial.

### Study examining mechanisms of intervention rather than effect or benefit

An additional study, Castellanos et al. [24], is worth mentioning. As previously stated, we first assumed that it studied effect or benefit but, on closer scrutiny, it became clear that it did not claim directly this but that it dealt with other relevant clinical issues, namely the question whether the neurophysiological measurements of brain activity (“entropy” and “complexity”) before and after treatment were linked to the clinical state of the subjects. To study this, the authors used a healthy control group for comparing their baseline values to the baseline values of the cases, to see if brain “entropy” and “complexity” were different in the two groups. After a neuropsychological rehabilitation program, a comparison was made again with the previously obtained baseline values of the healthy untreated group to see if the study subjects now resembled more the healthy control group than they did at baseline. The results were measured against information on activity of daily living. In other words, our interpretation was that the authors tested if the brain function issues that they addressed through intervention had a clinical value.

B. **Studies potentially able to detect effect or benefit of Functional Neurology**

*Daubeny* et al. [21] (Table 2, row 3): The best study, in terms of methodological quality, still had a quality score of only 4/9 (44%). In this randomized controlled trial with a sham treatment, the “blind spot” was measured before and after joint manipulation and found to change in a particular pattern. However, neither reliability nor reproducibility of the measurement of the “blind spot” were tested within the article or reported as reproducible or reliable on the basis of other studies. For this reason, it is not known if the findings in the present study can be trusted or if the findings could be fluctuating in a meaningless manner. Another problem is that the authors failed to describe clearly that its study subjects did not have a special interest or preconceived ideas in relation to manipulative therapy and the “blind spot”, as the origin of the study sample was unreported. This is important if study subjects could have been able to, willfully, change their visual reporting during the experiment. Further, as the validity of the “blind spot” as a neurological test with the ability to change with manipulative therapy has been questioned [8, 11, 13, 27], blinding in all possible ways is particularly important, i.e. also of the statistician, which was not described in the report. Therefore, although this study is a randomized controlled trial, it presents major methodological issues that potentially affect the validity of the reported results.

*Leisman & Melillo* [22] (Table 2, row 4): This study, 2/8 (25%), failed to report the use of a blind assessor and, therefore, it is not clear if the outcome could have been positively influenced or further aggravated by the absence of information on reproducibility of the collected data. Also, it was not reported if all study participants stayed to the end or even if they were all included in the final analysis. Although this study, apparently, reported on two randomized controlled trials consisting of one group of children with ADHD receiving or not receiving an intervention and a second group of “normal” age-matched children also receiving or not receiving (the same) intervention, we were unable to interpret the results. In fact, the authors did not clearly explain the results that were cryptically presented in a table and a figure and it was not clear to us exactly how comparisons were made between the four groups. Other methodological quality issues appeared when we completed our quality checklist.

*Carrick* et al. [17] (Table 2, row 8): Another report, 2/8 (25%), consists of two studies, containing analyses taking into account posturographic reactions in asymptomatic subjects, who were subjected to *whole-body rotations* in different planes. Eight and six outcome variables respectively were tested before and after the interventions at three different time intervals but there was not a control group that received no intervention. Admittedly, it would be difficult to make comparison to a sham *whole-body* intervention. For this reason, it would have been suitable to compare intervention to some other type of control in order to see if the tested intervention had some benefit. It would, also, have been possible to compare “correct” to “incorrect” intervention, to see if study subjects reacted differently to these in a logical manner.

In fact, when scrutinizing the research design, it gave the impression that this was the purpose, i.e. to compare “correct” to “incorrect” intervention. The study subjects were originally classified in relation to their different postural types in relation to “pitch” and “yaw”, i.e. the preferred position related to the head position when standing on a “perturbing foam cushion” with their eyes closed and the head rotated (“yaw”) and the head extended or flexed (“pitch”). Study subjects were divided into four groups according to their “pitch” and “yaw” predominance, after the foam cushion test, i.e. head in flexion, head in extension, head rotated to the left, head rotated to the right, and the various combinations of these.

Intervention was provided to all these four subgroups but not in the same way. The intervention was stated to be different in relation to the directions of “pitch” and of “yaw”. Thus, the study subjects were randomly allocated to receive a treatment (i) in the same directions as their preferred “pitch/yaw” postural reaction at baseline; (ii) in the same direction as their “pitch” position but opposite to their “yaw” position; (iii) in the opposite direction as their “pitch” position but in the same as their “yaw” position; or (iv) in totally opposite directions.

Presumably, although we did not understand the explanations of why this was done and what was expected, this design could be used to analyze if study subjects in the different groups would react differently on intervention, according to whether the classification and the intervention (i) matched, (ii) and (iii) matched partially, or (iv) did not match (as explained above). This sort of analysis would (perhaps) be able to provide information on whether the rationale for the intervention was correct or not. In addition to our checklist items, we noted that there were far too few study subjects in these two studies for the large number of tests and far too few values in each cell to allow for meaningful statistical analysis in the two reported studies. Also there was no report on the reproducibility of the “pitch” and “yaw” findings, meaning that the before-after measurements could be fluctuating regardless of intervention.

Further, the authors provide a very detailed and, in our opinion, confusing results section. This makes it difficult to understand if they actually tested whether the various intervention strategies based on the “pitch/yaw” classification resulted in different results for their many outcome variables. Only in the second study (p.355 l.15) is one of the stimulation groups mentioned. We were therefore confused as to whether the authors ignored the results of the matched intervention and only studied the change over time for different types of interventions and for different types of “pitch/yaw” classifications. This approach would in fact correspond to the study design of an outcome study. In other words, it looked to us as if they simply compared the baseline measurements at the three follow-up time points (i) for all treated subjects according to their base-line classification (p.346 under “Research Questions, l.4”) regardless if their treatment was matched to their classification or not, and (ii) for all four combinations of body rotation stimulation (p.346 under “Research Questions, l.5”) regardless if the classification group was treated in a “matched” manner or not. If we assume this to be the case, this confusing and complicated report would not have used its clever design to its full potential. This study also fails in other methodological aspects, as reported in Table 2.

*Sullivan* [18] (Table 2, row 9): This author reported on a prospective case series, where the clinician was also the assessor of the treatment outcomes, which reached a quality score of 2/8 (25%). This study seems to reflect the work of a clinician who has tested an original neurophysiological theory in his clinical practice. Some but not all study subjects (3/13) were subjected to a sham treatment but without random allocation, hence lacking a proper control group. Nevertheless, the results seem encouraging and might incite a future proper randomized control trial, as suggested by the author. Therefore, this study could be considered an interesting preliminary study, to see if the topic is worthwhile being pursued, but does not allow the author to deal with any effect or benefit of the tested intervention, i.e. pneumatic ear insufflation in the treatment of migraine.

### *Description of the evidence available in relation to clinical effect or benefit of Functional Neurology* (research objective 5)

Out of the nine studies that potentially dealt with effect or benefit of FN interventions, four were considered to, at least somewhat, be able to produce such answers. The quality scores of our quality checklist ranged from 25 to 44%, indicating an overall substantial risk of bias mainly in relation to: 1) blinding of study subjects, assessors and statisticians, and 2) concealment of random allocation. In addition, the outcome variables were never stated to be reliable or reproducible, making it reasonable to suspect that the reported results could be attributed to their inherent variability.

In light of these methodological short comings, we did not consider the results of these studies dependable. Therefore, we found no acceptable evidence that could support the notion that the FN approach has an effect or a benefit on the supposed indications tested, whether this was done on symptomatic or asymptomatic subjects. For this reason, the results of the various studies were not reported or illustrated as initially planned.

## Discussion

Out of a total of 168 texts published from 2011 to 2016 in the FNRE journal, 36 were identified as *research studies* in general, but only four could be classified as *clinical research studies* potentially investigating FN effect or benefit. A total of nine articles, five from the FNRE journal plus four from three other scientific journals (identified through the journal FNRE), were included for further description and analysis.

Due to design and methodological issues, no acceptable scientific evidence was found in relation to effect or benefit of various FN interventions. This was the case for studies on symptomatic children, mainly suffering from neurodevelopmental disorders, for symptomatic adults, suffering from migraines or traumatic brain injury, and also for asymptomatic adults on whom balance or “blind spot” changes after FN interventions were investigated. All had the hallmark of FN, i.e. targeting different parts of the brain, but did not bring any evidence on indications for treatment or for the best match between condition and intervention.

### Considerations regarding the type of literature captured

The few *research studies* in general, i.e. covering FN topics or not, indicates that the authors who publish in this journal are more inclined to write discussion papers or narrative reviews than *research studies*. Further, within this small group of research articles, only a few were relevant for our review. Nevertheless, given the small percentage of *research studies*, it could be argued that research on FN has or has not been published extensively outside this journal. But, in a previous review, the present authors also noticed that only few scientific articles were easily available in general on the clinical aspects of FN but from a chiropractic perspective [1]. Thus, the present critical review also reveals this paucity of research evidence in relation to the effect or benefit of FN.

Obviously, academic inclined clinicians have a need to read and exchange. The FNRE journal seems to provide ample opportunities for this but there is an obligation on all scientific journals not only to discuss and claim but also to establish the basic scientific facts. This critical review failed to find robust evidence of the latter.

### Methodological considerations of reviewed studies

#### The study design

Several studies did not even include a control group and only one compared the intervention group with a sham intervention in a randomized controlled trial.

In studies without control groups, only *outcome* can be reported; thus it is not appropriate to talk about effect or benefit. The reasons for this are that studies without a control group, showing improvement after intervention, may indicate a true effect, a regression towards the mean, or simply the natural course of a disease that gets better on its own or has its ups and downs, but it is not known what. Thus, the scientific interpretation is usually only of a positive *outcome*, a potential benefit but certainly not of effect of intervention. This is the main reason why clinicians who see their patients improve cannot claim to have an “effect”, only hope there is, as there are no placebo groups possible in clinical practice.

Clearly, most of the authors of the articles included in the present review, as has been found before in another chiropractic research field [28], seem not to be completely familiar with the requirement to be meticulous about matching the research question to the correct research design.

#### The research methods

Each research design (e.g. surveys, clinical trials, population-based studies) has its specific requirements. These are based on logical rules that are well accepted in the scientific community, although errors and omissions are often observed in the research literature. The articles reported in this review were often non-observant of these rules, at least for those pertaining specifically to randomized controlled trials.

For randomized controlled trials, whether comparing treatment to a placebo procedure or another treatment, well established quality checklists exist, such as that used by the Cochrane collaboration [29]. We did not use this but extracted only few important points, as it was evident that the reviewed literature was deficient and that it would be meaningless to go into details. We conclude that the few items we selected were sufficient to point the reader in the right direction.

In sum, the major finding of this review was the lack of conventional use of research design and method in order to investigate any *effect* or *benefit* of the FN approach. Of serious concern was the lack of information regarding approval from an identifiable human research ethics committee. We also noted that although some did report conflicts of interest, some did not mention this aspect or seemed to do so incompletely.

### Methodogical considerations of own review

Searching one single journal had the advantage that we were unlikely to miss studies of interest. However, this does not mean that all the scientific literature on the FN field has been covered in the present review. Nevertheless, this was not our intent. The motivation to restrict the search to the FNRE journal was already evoked in the Introduction of this article. In fact, we have previously established that the FN literature was difficult to find [1]. The main reasons for this are that publications on this topic usually are not associated with the key word “functional neurology” and FN proposes so many treatment or intervention approaches (types of stimuli) for so many conditions that it would probably be impossible to design a relevant search equation to capture all the FN literature.

The quality checklist used in the review was specifically designed for our purpose but, potentially, other researchers might select other items to assess the methodological quality (including risk of bias) of the included studies. Nevertheless, given the problems relating to design and methodological issues discussed above, it is very unlikely this would affect the conclusion of the present review.

Also, we adopted a lenient approach for inclusion of the studies in our final analysis, selecting randomized and non-randomized studies, with or without proper control groups. A more stringent selection would have brought even less studies to discuss. This flexibility gave us the opportunity to address design and methodological issues in order to bring the reader, especially clinicians and health care students, some basic knowledge needed to effectively consume research reports. Not all health practitioners have adequate skills and experience in the reading of the research literature. This is despite the need to critically appraise the literature encountered during their career, even when such literature is promoted and produced by their colleagues. This is also true for FN, a movement within which research has a clinical and commercial component [30].

## Conclusion

We can conclude that the FNRE journal, with a special interest in FN, contains only few clinical research articles in this field. Further, it is clear that over five years and twenty-four issues of this journal, no methodologically sound studies on the effect or benefit of the FN approach were published. In order to find out if there is, in fact, other relevant documentation on the effect or benefit of FN, a critical review of the scientific publications of its founder, FR Carrick, apparently actively involved in research, may be able to fill in the gaps regarding the scientific state of FN.

## Appendix

### Items selected for the second part of the quality checklist (Table 2) and their rationale

**In relation to study subjects**:

**- Were study subjects stated to be unbiased (blind and naïve)?** The reason why it is important that subjects are blind to the nature of the experiment is that subjects may be influenced by their expectations to treatment outcomes. For the same reason and when this is not possible to blind the subjects, i.e. when the intervention is not compared to a sham intervention but to another intervention, subjects have to be at least naïve to the intervention they receive.

**- Was allocation to study groups stated to be randomized and concealed?** The random allocation and its concealment minimize the risk of selection bias.

**In relation to the experiment**:

**- Was the intervention well described?** A sufficient description of the investigated intervention is one key element that allows to replicate the study, especially when this is a new and/or multifaceted intervention.

**- Was the outcome measure reported to be reproducible or reliable?** An acceptable reproducibility and reliability are needed to ensure that the study results are not simply due to normal variations of the measures over time or due to intra and/or inter-examination variabilities when performing the measures.

**In relation to the assessment**:

**- Was the assessor stated to be blind to group allocation?** When not blinded, the assessor may be influenced by his/her wish to obtain better results in the intervention group compared to the sham/control group.

**In relation to analysis and data reporting**:

**- Was the statistician stated to be blind to group allocation?** This is for the same reason that the assessor has to be blind.

**- Were losses and exclusions reported or obvious in results, tables or graphs?** Reporting losses and exclusions, if any, allows to appreciate in which extent this could affect the reported results.

## Abbreviations

ADHD: attention deficit and hyperactivity disorders
FN: Functional Neurology
FNRE: Functional Neurology, Rehabilitation, and Ergonomics
